# Revolutionizing Bone Regeneration with Grinder-Based Dentin Biomaterial: A Systematic Review

**DOI:** 10.3390/ijms25179583

**Published:** 2024-09-04

**Authors:** Anna Olchowy, Cyprian Olchowy, Ireneusz Zawiślak, Jacek Matys, Maciej Dobrzyński

**Affiliations:** 1 Department of Experimental Dentistry, Wroclaw Medical University, Krakowska 26, 50-425 Wroclaw, Poland; ania.olchowy@gmail.com; 2 Collegium Medicum, Jan Dlugosz University in Czestochowa, Armii Krajowej 13/15, 42-217 Czestochowa, Poland; cyprian.olchowy@gmail.com; 3 Faculty of Biotechnology and Food Sciences, Wrocław University of Environmental and Life Sciences, Chełmońskiego 37, 51-630 Wroclaw, Poland; ireneusz.zawislak@upwr.edu.pl; 4 Oral Surgery Department, Wroclaw Medical University, Krakowska 26, 50-425 Wroclaw, Poland; 5 Department of Pediatric Dentistry and Preclinical Dentistry, Wroclaw Medical University, Krakowska 26, 50-425 Wroclaw, Poland; maciej.dobrzynski@umw.edu.pl

**Keywords:** bone regeneration, bone grafting, bone substitutes, demineralized dentin matrix, autogenous tooth graft, tooth grinder, dentin grinder

## Abstract

Bone tissue regeneration is a critical aspect of dental surgery, given the common occurrence of bone resorption leading to alveolar bone defects. The aim of this paper was to conduct a systematic review to provide a comprehensive summary of the evidence regarding the regenerative properties of dentin biomaterial. This systematic review was conducted through comprehensive searches in the PubMed, Scopus, and Web of Science databases, as well as an extensive exploration of the gray literature sources, including WorldCat, The New York Academy of Medicine Library, and Trip Database, following the established PRISMA protocol. Keywords such as tooth, dentin, grinder, and autograft guided the search, with a focus on a standardized procedure involving dentin grinders within laboratory, experimental, and clinical settings. Initially, a pool of 1942 articles was identified with 452 duplicates removed. An additional 1474 articles were excluded for not aligning with the predefined topics, and three more were excluded due to the unavailability of the full text. Ultimately, 13 articles met the strict inclusion criteria and were included in the review. The chemical composition of the dentin particles was similar to natural bone in terms of oxygen, carbon, calcium, phosphorus, sodium, and magnesium content, as well as in terms of the Ca/P ratio. In addition, the dentin also contained amide I and amide II structures, as well as aliphatic and hydroxyl functional groups. The chemically treated dentin was free of microorganisms. The dentin had characteristic tubules that opened after chemical treatment. At the cellular level, dentin released bone morphogenetic protein 2, induced significant cell growth, and stimulated the reorganization of the fibroblast cytoskeleton. Most clinical studies have focused on alveolar bone regeneration. After the transplantation of demineralized dentin particles, studies have observed new bone formation, a reduction in residual bone, and an increase in connective tissue. Clinical reports consistently indicate uncomplicated healing and recovery post-transplantation. However, there is a notable gap in the evidence concerning complication rates, patient-reported outcomes, and the presence of pro-inflammatory factors. In conclusion, dentin biomaterial emerges as a versatile bone substitute, demonstrating high biocompatibility and ease of acquisition. The preservation of its internal structure containing organic matter and growth factors enhances its potential for effective bone regeneration. Particularly, in dental surgery, dentin-derived materials present a promising alternative to traditional autologous bone autografts, offering the potential to reduce patient morbidity and treatment costs.

## 1. Introduction

**Figure 1:**
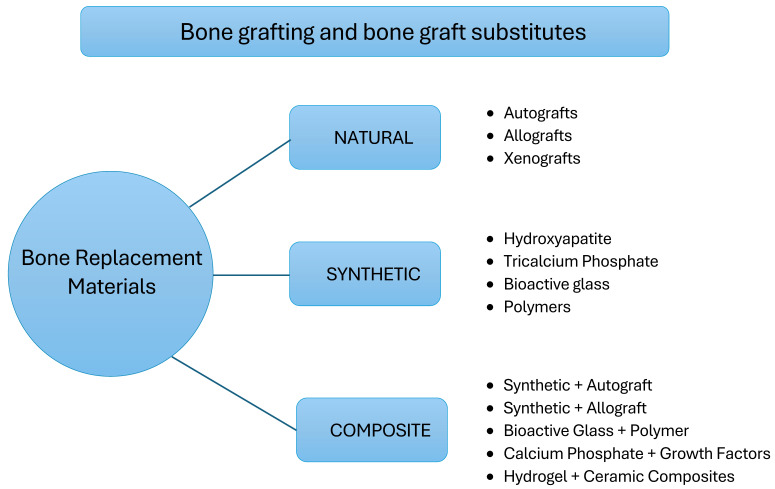
Classification of bone replacement materials [1,2].

Bone tissue regeneration is a critical aspect of orthopedic and dental surgery. Conditions requiring bone tissue regeneration can be caused by trauma, infections, malignant lesions, or congenital defects or can develop as a consequence of applied treatment, such as following radiotherapy of malignant lesions [1,2]. In dentistry, the occurrence of bone resorption leading to alveolar bone defects surpasses 90% and depends on patient demographics, oral hygiene practices, and general health [3]. Bone grafting and bone graft substitutes are frequently employed to provide structural support, protect from bone loss, secure capacity for future prosthetic implantation, and facilitate bone healing. A variety of bone grafts are available and are classified according to their origin into natural, synthetic, and composite categories. Natural bone graft materials encompass autogenic allogeneic and xenogeneic sources, while synthetic options include hydroxyapatite, tricalcium phosphate, bioactive glass, and polymers. Composite bone graft materials represent a hybrid of natural and synthetic components. Each type of bone graft material presents a distinct set of advantages and disadvantages [4] (see Figure 1). In the USA, the use of bone graft procedures has been on a downward trend over time. During the period from 2000 to 2003, autogenous bone grafts accounted for 83% of the procedures, while artificial bone grafts constituted 17%. Nonetheless, with the aid of contemporary advancements and novel surgical techniques, it is now feasible to extend the eligibility criteria for older patients, reduce the treatment burden, and shorten the duration of procedure-related hospitalization [4].

Autologous bone refers to bone tissue harvested from the same patient, minimizing the risk of immune rejection and preserving robust osteogenic, osteoinductive, and osteoconductive properties [5]. In contrast to allogeneic and xenogeneic grafts, autologous bone exhibits lower resorption rates [6]. Despite these advantages, the harvesting of autogenous bone carries a potential risk of donor site morbidity [7], prompting a recent trend toward substitutive grafts [4]. Allogeneic and xenogeneic bone grafts involve the use of bone material from one individual to another within the same species or between different species. Although they are associated with higher resorption rates and an increased risk of immunologic reactions and disease transmission, these grafts eliminate donor site morbidity, thereby reducing the treatment burden on the patient [8]. Given the limited efficacy of natural bone grafts, various synthetic bone substitutes have been investigated in clinical practice as alternative options. The objective was to offer an economical graft that closely mirrors the structure and strength of human bone while facilitating new bone formation [9]. Despite their ability to replicate the structure and composition of natural bone, be customized, and eliminate disease transmission, synthetic bone substitutes may provoke a foreign body response. Additionally, their constrained biological activity and absence of cellular components found in natural bone can result in reduced efficiency in the regeneration process [10]. For these reasons, the focus has shifted toward osteoinductive materials containing growth factors, such as the demineralized bone matrix and bone morphogenetic proteins. Clinical outcomes may vary due to differences in osteoinductive properties, and considerations arise regarding the concentration of growth factors, tissue source, and potential variations in production process parameters. The high cost of the procedure further serves as a limiting factor [11].

**Figure 2:**
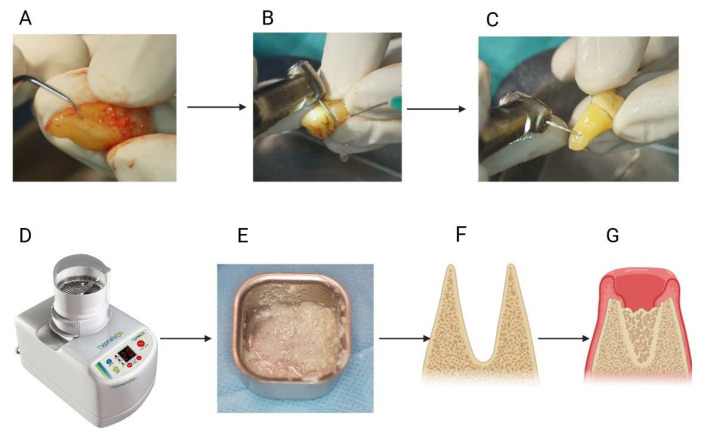
Diagram of the specimen preparation and clinical application process: (**A**,**B**) debridement of the third molar root following extraction; (**C**) removal of the tooth crown before grinding; (**D**) use of the Smart Dentin Grinder™ apparatus (KometaBio Inc., Cresskill, NJ, USA); (**E**) graft material post-grinding; (**F**) image of the alveolar socket before grafting; (**G**) image of the alveolar socket after grafting.

Human dentin materials from extracted teeth were traditionally deemed unsuitable for use as a graft in bone regeneration. The first clinical case report documenting the utilization of patient-owned dentin for bone augmentation was published in 2002 [12]. Subsequently, researchers have started to develop diverse processing techniques for non-functional teeth, transforming them into a viable native resource for bone regeneration. Pang et al. [13] conducted a comparative study assessing the efficacy of autogenous tooth graft material and anorganic bovine bone. The teeth were mechanically crushed into particles ranging between 300 and 800 µm in diameter. Subsequently, the particulate teeth underwent a series of processes, including washing, defatting, decalcification, and lyophilization. The resulting autogenous demineralized dentin matrix, as well as the anorganic bovine bone, were grafted into extraction sockets post-tooth extraction to address bone defects. The utilization of an autogenous demineralized dentin matrix demonstrated effectiveness comparable to that achieved with anorganic bovine bone. Both groups displayed favorable wound healing, comparable implant stability, and histologically confirmed new bone formation. Similar results were reported in other studies [14,15] (Figure 2).

The aim of this project was to conduct a systematic review to provide a comprehensive summary of the evidence concerning the regenerative properties of dentin biomaterial. Understanding the impact of dentin as a biomaterial, including at the molecular level, is crucial for clinicians. This knowledge can help clinicians in selecting the appropriate type of dentin-derived material based on the specific clinical scenario.

## 2. Materials and Methods

### 2.1. Focus Question

With a particular emphasis on the outcomes associated with the utilization of a standardized grinding protocol utilizing a dentin grinder for the preparation of dentin biomaterial in various settings, including laboratory, experimental, and clinical contexts, what is the extensive molecular evidence concerning the regenerative attributes of dentin biomaterial?

### 2.2. Protocol

**Figure 3:**
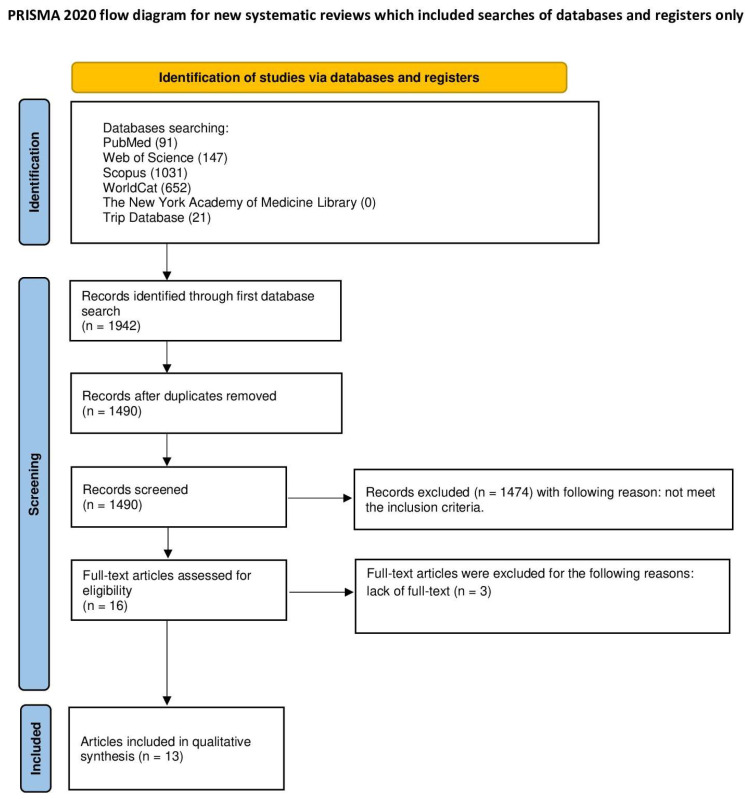
PRISMA flowchart.

The selection process for articles in the systematic review was carefully outlined following the PRISMA flow diagram (Figure 3). The systematic review was registered on the Open Science Framework at the following link: https://osf.io/zj7p8 (accessed on 20 August 2024).

### 2.3. Eligibility Criteria

The inclusion and exclusion criteria were developed according to the Population, Intervention, Comparison, Outcomes, and Study Design (PICOS) framework [16]. All inclusion and exclusion criteria are listed in Table 1. Our search was restricted to articles published in English; nevertheless, no limitations were applied regarding the publication timeframe or geographical scope. The search specifically focused on a specific dentin grinding method utilizing a dentin grinder in the human model. This limitation was justified by the availability of a standardized grinding procedure outlined in the manufacturer’s instructions. The grinder is programmable to generate particles within a specified size range of 300 μm to 1200 μm, ensuring standardization and reproducibility across all studies [17].

### 2.4. Information Sources, Search Strategy, and Study Selection

In May 2024, a thorough and systematic electronic exploration unfolded across prominent scholarly databases, including PubMed, Cochrane, Web of Science, and Scopus. In addition, the gray literature sources such as WorldCat, The New York Academy of Medicine Library, and Trip Database were searched. To ensure a comprehensive search of the relevant literature, the search strategy used MeSH terms, such as “tooth”, “dentin”, and “autograft”, and also included non-MeSH keywords, such as “grinder”. The search criteria were carefully designed, focusing on a strategic combination of the mentioned keywords. For PubMed, we used ((tooth[Title/Abstract]) OR (dentin[Title/Abstract])) AND ((grinder[Title/Abstract]) OR (autograft[Title/Abstract])). For WoS, we used AB = ((tooth OR dentin) AND (grinder OR autograft)). For Scopus, we used (TITLE-ABS-KEY (tooth) OR TITLE-ABS-KEY (dentin)) AND (TITLE-ABS-KEY (grinder) OR TITLE-ABS-KEY (autograft)). For WorldCat, we used ((tooth) OR (dentin)) AND ((grinder) OR (autograft)). For The New York Academy of Medicine Library, we used ((tooth) OR (dentin)) AND ((grinder) OR (autograft)). For Trip Database, we used ((tooth) OR (dentin)) AND ((grinder) OR (autograft)). The search methodology strictly adhered to the guidelines set forth in the Preferred Reporting Items for Systematic Review and Meta-analysis (PRISMA) statement [18]. Supplementary searches were conducted in journals pertinent to oral surgery and periodontology. Subsequent to the database search, a meticulous and exhaustive literature review was systematically carried out to identify any papers initially deemed potentially irrelevant to this study. Inclusion considerations were strictly reserved for articles with full-text versions.

### 2.5. Data Collection Process and Data Items

During the initial stage of study selection, the authors independently reviewed the titles and abstracts of each study. Any discrepancies in the inclusion or exclusion of articles were thoroughly discussed among the reviewers to ensure consensus. Three reviewers (C.O., A.O., I.Z.) autonomously performed data extraction from articles that satisfied the predefined inclusion criteria. The gathered information was then meticulously recorded in a standardized spreadsheet for systematic organization and analysis.

### 2.6. Quality Assessment

Two independent assessors (C.O., I.Z.) systematically evaluated the methodological rigor of each study to decide on the inclusion of the studies. In the event of a disagreement between reviewers regarding the inclusion of an article, a third reviewer was consulted to resolve the dispute and make the final decision. For the quality assessment, a set of critical appraisal tools was developed by the Joanna Briggs Institute (JBI) [19].

To assess inter-rater reliability, Cohen’s kappa test was conducted. The results of Cohen’s kappa test indicated perfect agreement among the reviewers, with a kappa value of 1.0, demonstrating 100% consistency in their assessments.

## 3. Results

### 3.1. Study Selection

Initially, 1942 articles were identified, with 452 duplicates removed. A further 1474 articles were excluded for not meeting the predefined topics, and three were excluded due to lack of access to the full text. In the end, 13 articles met the strict inclusion criteria and were included in the review. These comprised three in vitro studies [20,21,22] and ten human studies [14,15,17,23,24,25,26,27,28,29]. The human studies involved a total of 127 patients who were subjected to the procedure with a smart dentin grinder, with three case reports and studies ranging from nine to fifty-two patients.

### 3.2. Molecular Aspects of Ground Dentin Grafts

Molecular aspects were exclusively evaluated in three studies [20,21,22]. None of the studies focused solely on clinical outcomes. Ten studies presented results on the chemical composition or morphology evaluation of the material before grafting, as well as histomorphometric evaluation of bioptates obtained during implantation, alongside clinical data [14,15,17,23,24,25,26,27,28,29]. The understanding of the molecular aspects in studies involving bone tissue regeneration with ground dentin grafts is pivotal for the integration of a biomaterial into clinical practice. This aspect was explored through in vitro studies, morphometric analyses, and histological studies. This aspect was investigated through in vitro studies using the FTIR, ELISA, and XTT tests. The characteristics of the included studies are presented in Table 2.

#### 3.2.1. Chemical Composition

Four studies provided insights into the composition of dentin after grinding. The analysis conducted by Khanijou et al. [21] showed that tooth-derived material comprised oxygen (50.6%), carbon (22%), calcium (15.7%), phosphate (9.8%), sodium (1.4%), magnesium (0.6%), and a calcium-to-phosphate ratio (1.83), calculated based on the atomic percentage. Sarna-Boś et al. [22] conducted a similar analysis of dentin composition, although with a different presentation of the results. Sarna-Boś et al. [22], on the basis of icosahedral analysis of the description of functional groups using FTIR spectrometry, showed the presence of phosphate ions, carbonate anions, and amide I and amide II structures, as well as aliphatic and hydroxyl groups. Material obtained from premolars was characterized by a higher content of phosphate ions, carbonate anions, amides, and hydroxyl groups compared to material obtained from incisors and canines. However, a higher content of aliphatic groups was observed in incisors compared to other tooth types. Moreover, an analysis of the elemental composition of dentin by ICP OES and SEM-EDX was performed. The obtained conclusions were significant, as they demonstrated that all four groups of human teeth (incisors, canines, premolars, molars) showed a similar chemical composition. The overall composition included calcium (276.4 ± 9.7 g/kg), magnesium (5.2 ± 0.9 g/kg), sodium (2 ± 0.1 g/kg), phosphate (147.6 ± 4.9 g/kg), and a calcium-to-phosphate ratio of 1.9 ± 0.02. Dłucik et al. [24] checked the elemental composition of crushed particles with three different devices (BonMaker; Korea Denta Solution Co., Ltd., Busan, Republic of Korea, Tooth Transformer, SRL, Milan, Italy, Smart Dentin Grinder, KometaBio Inc., Cresskil, NJ, USA) and compared them to the control dentin. The results obtained from energy-dispersive X-ray analysis showed significant differences. In the dentin specimen, the primary components were carbon, oxygen, phosphorus, and calcium. The notably high levels of calcium and phosphorus indicated the mineral content. In Smart Dentin Grinder specimens, calcium dominated, constituting over 50% of the elements sampled. Carbon, oxygen, and phosphorus were also present, with percentages similar to those found in dentin. The Raman spectra also differed. The most pronounced band for all devices appeared in the region centered at 957 cm^−1^, attributed to the inorganic component. However, there were variations between devices in terms of the Raman signal in the wave number region of 2843–3005 cm^−1^, associated with the organic component. Finally, Del Canto-Díaz et al. [27] using electron microscopy confirmed an atomic composition and a calcium-to-phosphate ratio similar to that of bone.

#### 3.2.2. Evaluation of Cellular Responses

The evaluation of cellular responses was carried out in two in vitro studies. Bianchi et al. [20] evaluated the impact of different dentin derivatives at the cellular level in a study with human periodontal ligament fibroblasts. They reported that mineralized dentin induced significant cellular growth in comparison to deproteinized bovine bone at 7 days. In addition, fibroblasts treated with mineralized dentin exhibited a thickening of the cellular membrane and showed strong vinculin and integrin signals at 72 h, while the actin signal remained constantly expressed during the entire follow-up. In their study, actin, vinculin, and integrin were considered markers, indicating the reorganization of the cytoskeleton. The observed differences in adhesion and proliferation between TT and DDP suggest an important role in the composition of these materials in cellular responses. TT, being a richer source of collagen and bone morphogenetic protein (BMP), exhibited stronger adhesion and proliferation effects compared to DDP, which was devoid of them. The presence of collagen in TT may influence better adhesion of gingival fibroblast cells (hPLFs), as collagen is known to promote cell–substrate interactions. Additionally, the presence of BMP in TT may affect proliferation processes, as BMP is a cell-inducing factor. In another study, Khanijou et al. [21] investigated bone morphogenetic protein-2 (BMP2) from graft material. BMP2 belongs to the transforming growth factor beta (TGF-β) superfamily and plays a key role in osteogenesis. This study observed that both tooth-derived material and allograft began releasing BMP2 after graft preparation with a subsequent gradual decline observed until day 10. BMP2 was significantly more abundant in the release from dentin material compared to allograft and was measured at day 5 (162.81 ± 9.03 pg/mL vs. 132.15 ± 3.85 pg/mL; *p* < 0.05) and day 10 (212.53 ± 9.11 pg/mL vs. 175.75 ± 2.25 pg/mL; *p* < 0.05). Nevertheless, there was a lower increase in the migration of human fetal osteoblastic cells when cultured with dentin material compared to allograft (44.4% vs. 59.2%) on day 3.

### 3.3. Morphology

Dentin material underwent evaluation using various microscopy techniques immediately after grinding. Based on SEM analysis, the researchers consistently observed a comparable size range of dentin particles post-grinding, ranging from 300 μm to 1200 μm [21,23,26,27,28], 250 to 1200 μm [14], or 300 μm to 1300 μm [24]. Sarna-Boś et al. [22] evaluated chemically untreated dentin particles using scanning electron microscopy (SEM) and reported that dentin particles appeared crumbled but not crushed. These particles contained cylindrical prisms of enamel, dentin with characteristic tubules, and innervated radicular pulp. The peak maximum for pores was observed at a diameter of about 55 μm. The total porosity did not exceed 29%, and the pore area was 1.89 m^2^/g. Based on SEM images, Khanijou et al. [21] reported that the crushing force of the dentin grinder opened the dentinal tubules, but their visibility and homogeneity became apparent only after chemical treatment. Before the chemical treatment, dentin particles showed a regular pattern of dentinal tubules with occluded organic contents between them, resulting in an unclear tubular pattern. After chemical treatment, the area surrounding the dentinal tubules exhibited an irregular and coarse surface structure. Although the dentinal tubules seemed more blocked before chemical treatment, the surrounding area appeared smoother. The conclusion drawn was that chemical treatment played a crucial role in revealing the homogeneity of dentinal tubules and influencing surface characteristics. Binderman et al. [15] present the progressive opening of tubuli openings during cleansing and their wide opening at 10 min. Dłucik et al., 2023 [24] compared demineralized dentin produced with three different devices (BonMaker, Tooth Transformer, Smart Dentin Grinder). SEM showed important differences in the size and shape of granules. Particles prepared with a Smart Dentin Grinder were larger and had sharp margins. The particles’ surfaces were mostly flat, occasionally displaying linear reliefs. At higher magnification, these reliefs exhibited a lamellar shape, featuring dentinal tubules and orifices. Some particles displayed deeper and orderly rows, especially on their lateral surfaces.

In a study by Del Canto-Díaz et al. [27], the prismatic and irregular appearance of dentin fragments was observed using an optical microscope.

### 3.4. Histomorphometric Outcomes

The study carried out by Cervera-Maillo et al. [23] selected 10 healthy patients for the treatment of their atrophic edentulous mandible. After their teeth were ground, particulate dentin was placed in empty sockets and bone defects after teeth extractions. Histomorphometric analysis showed the continuous formation of new bone (16.3 at 3 months vs. 59.4 at 12 months), a decrease in residual bone (37.1 at 3 months vs. 15.6 at 12 months), and a decrease in the amount of connective tissue (46.6 at 3 months vs. 25 at 12 months). Illustratively, initially, the teeth particles were immersed in a developing connective tissue with minimal new bone formation. Subsequently, small particles of dentin became integrated into new, immature bone. Then, a substantial amount of bone formation surrounding the tooth particles became apparent. Finally, a new bone structure with incorporated dentin particles was observed. A randomized controlled trial conducted by Santos et al. [14] enrolled 52 patients (66 implants). Primary implant stability at baseline and secondary stability at 2 months after implant placement were similar between mineralized dentin and xenograft. The percentage of newly formed bone was higher for mineralized dentin than for xenograft (47.3% vs. 34.9%; *p* < 0.001), while the percentage of the residual graft was lower for mineralized dentin than for xenograft (12.2% vs. 22.1%; *p* < 0.001).

Four studies examined the histology of bioptats collected during implantation. In the study by Pohl et al. [26], the presence of new bone tissue that was in direct contact with dentin particles was noted. Additionally, the presence of osteoblasts was confirmed using specific antibodies. In the study conducted by Binderman et al. [15], dentin with its tubules was observed, surrounded by a newly formed bone matrix. In Dłucik et al.’s study [28], histological biopsies were taken 4 to 5 months after grafting ground in the alveolar bone in the maxilla. Examination of the images revealed that the majority of dentin fragments were enveloped by newly formed bone, while some fragments remained enclosed by soft connective tissue. Stained images displayed osteocytes within the bone tissue and osteoblasts on its surface, clearly indicating the distinction between newly formed and fully mineralized bone. No signs of inflammation or other pathological processes were noted.

Histometric analysis showed a significant difference in osteoid thickness between the groups. The osteoid thickness was 8.35 µm in the Bio-Oss group compared to 13.12 µm in the autogenous tooth bone graft (AutoBT) group. This result suggests that there are noticeable changes in bone mineralization between the groups. The reduced osteoid thickness in the Bio-Oss group may indicate a more advanced mineralization process compared to the AutoBT group. In addition, computed tomography (CT) analysis showed no significant differences between the groups in total bone volume, new bone volume, and new bone mineral density. There was, however, a significant difference in trabecular thickness, which was 0.07 µm in the Bio-Oss group compared to 0.08 µm in the AutoBT group [29].

### 3.5. Bacteriological Purity

Three studies provided information on bacterial purity. Binderman et al. [15] reported that their bacteriological test showed no bacterial growth after a 10 min treatment with the cleanser, while Khanijou et al. [21] revealed that the initial colony forming unit (CFU) per 0.1 g was 2.3 × 10^6^ before chemical treatment. However, after the chemical treatment, no microorganisms were detected. Dłucik et al. [24] confirmed obtaining negative results in microbiological testing.

### 3.6. Clinical Outcomes

Clinical outcomes were limited, with a focus on radiographic imaging for evaluating tissue density and in vivo structural changes in the examined bone tissue. Few studies addressed complication rates and patient-reported outcomes.

#### 3.6.1. Bone Tissue Regeneration

The study by Pohl et al. [26], involving 13 patients, utilized radiographic comparative analysis in order to evaluate dimensional ridge changes 4 months after tooth extraction and immediate grafting with mineralized dentin particulate autograft and chopped platelet-rich fibrin. Extraction sockets with up to 2 mm of missing buccal bone in the coronal aspect were compared to the lingual bone. At the end of the study, cone beam computed tomography revealed an average mid-buccal bone height gain of +1.1% and a mid-lingual height gain of 5.6%.

Binderman et al. [15] shared their broad experience in grafting the demineralized dentin matrix; however, they presented two challenging case reports in detail. In case 1, the surgically removed tooth exposed the distal root surface of the neighboring tooth. A follow-up after 4 months revealed a normal pattern of marginal gingiva around the neighboring tooth, with normal probing depths of 1–2 mm. On the X-ray, new bone and dentin were integrated, fully restoring the extraction site and supporting adjacent teeth. In case 2, two teeth were removed due to poor periodontal attachment, increased mobility, and bone loss. Following extraction, granulation tissue was cleared, exposing bone walls, and dentin particles were inserted into sockets and covered with a platelet-rich fibrin membrane. Implants were placed after a 2-month interval. A 2-year follow-up X-ray showed highly radiopaque bone integrated into the implants providing robust support.

Del Canto-Díaz et al. [27] evaluated the outcomes of their split-mouth study conducted on nine patients requiring the extraction of two single-rooted teeth and subsequent rehabilitation with osseointegrated implants. The condition of post-extraction sockets after filling with autologous dentin material was compared to this without filling with any material. The dentin graft exhibited a lower mean vertical bone loss (4.2% vs. 16.87%) and a reduced mean horizontal loss of buccal cortical bone at 16 weeks (0.16 mm vs. 2.22 mm; *p* = 0.067) compared to no graft. Additionally, densitometric analysis of the coronal alveolar area indicated higher bone density for the dentin graft compared to no graft (922.68 ± 250.82 HU vs. 564.35 ± 288.73; *p* = 0.045).

Matsuzawa et al. [25] reported a case involving the repair of a unilateral maxillary alveolar cleft during mixed dentition. At the 6-month follow-up X-ray, the grafted demineralized dentin was identifiable as radiopaque granules, distinguishable from the original bone by both its structure and radiopacity. After 6 years, the graft composed of demineralized dentin from autogenous primary teeth was entirely replaced by bone, without affecting tooth formation or impeding spontaneous tooth eruption.

In the split-mouth case report by De Biase et al. [17], the X-ray results indicated less bone loss at the site treated with a demineralized dentin matrix compared to the control site. Specifically, when measuring the distance between the cementoenamel junction (CEJ) and the bone peak, the control site exhibited a reduction of 0.94 mm, whereas in the grafted site, the reduction was 2.32 mm. The clinical probing depth decreased by 1 mm in the grafted site, while no changes were observed in the control site.

Dłucik et al. [28] reported the absence of significant bone resorption during the follow-up period, irrespective of the method employed.

#### 3.6.2. Periprocedural and Long-Term Complications

In a randomized controlled trial conducted by Santos et al. [14] with 52 patients, a comparison between mineralized dentin and xenograft resulted in the lack of significant differences in terms of bleeding, marginal bone loss, the amount of keratinized gingival width, and the occurrence of peri-implant mucositis. In a study by Pohl et al. [26] involving 13 patients who received mineralized dentin with platelet-rich fibrin to fill post-extraction sockets, there were no observed signs of inflammation or fibrosis formation around the autologous augmentation material. Several other studies reported an uncomplicated healing process and recovery following the surgical procedure and grafting [15,25], with one study reporting no clinical and radiographic signs of complications at six months post-procedure [17]. In all alveolar bone regeneration and sinus lift procedures, Dłucik et al. [24] reported the absence of significant complications during the recovery period, ensuring uneventful healing.

#### 3.6.3. Patients’ Reported Outcomes

Only one study focused on patient-reported outcomes. Among the 52 participants in the study by Santos et al. [14], patients reported significantly lower pain experience the next day after the procedure with demineralized dentin in comparison to xenograft (*p* = 0.014). Furthermore, drawing from the aggregated outcomes across groups employing various graft preparation devices, Dłucik et al. [28] concluded that none of the patients reported additional pain or discomfort.

### 3.7. Quality Assessment

Three case reports were evaluated with a checklist. One case report received a maximum score of 8 points [25], while two others received 6 points [17,25]. Only one case series was included [27] that received a maximum score of 10 points. Only one cohort study was included [23] with 8 points out of 11 possible. Of the two randomized controlled trials, one had a low risk of bias with 11 points out of 13 possible [14], while the other had a moderate risk of bias with 7 points out of 13 possible [29]. The risk of bias in three in vitro studies was not assessed due to the lack of a relevant checklist [20,21,22]. Table 3 presents the scoring for the evaluated studies.

## 4. Discussion

Our systematic review aimed to provide a thorough insight into the potential application of ground dentin grafts for bone tissue regeneration. Commencing with a focus on molecular aspects explored in both in vitro and histomorphometric studies, progressing through experimental models, and culminating in the assessment of clinical outcomes, this review bridges the gap between understanding the underlying biological mechanisms and evaluating clinical effectiveness. Various studies in the literature have demonstrated the significant role of autologous dentin in bone reconstruction [14,15]. In a randomized controlled trial, Santos et al. [14] compared autogenous mineralized dentin to xenograft granules for ridge preservation in delayed implantation. The percentage of newly formed bone within the mineralized dentin matrix was significantly higher than that observed with xenograft (47.3% vs. 34.9%; *p* < 0.001), with a lower percentage of residual graft (12.2% in mineralized dentin matrix vs. 22.1% in xenograft; *p* < 0.001). No significant differences were identified in clinical, radiographic, and patient-related outcomes. The mineralized dentin matrix was prepared using a standardized protocol with a grinder generating particles with diameters between 250 and 1200 μm. Although various protocols were employed by other researchers, potentially influencing the results, the outcomes remained promising despite the divergence in dentin biomaterial processing methods [15].

Bone regeneration is a crucial aspect of oral and maxillofacial surgery, particularly in procedures like placing dental implants, bone grafting, and the surgical treatment of periodontal diseases [30]. This process involves the restoration of bone tissue in areas where bone has been lost due to disease, trauma, or tooth extraction [30]. Advances in regenerative techniques, such as guided bone regeneration (GBR), the use of biomaterials, like bone grafts (autografts, allografts, xenografts), and growth factors, such as platelet-rich plasma (PRP) or bone morphogenetic proteins (BMPs), have significantly enhanced the ability to promote bone healing and regeneration [30,31,32,33]. Among these, autologous dentin grafts, derived from a patient’s own extracted teeth, offer unique advantages [25,26]. These grafts are biocompatible, eliminating the risk of immune rejection, and they closely mimic the natural composition of bone, facilitating more efficient integration and regeneration. Additionally, using a patient’s own dentin reduces the need for external donor materials, making the process more cost effective and reducing the risk of disease transmission [14,29]. These methods not only reconstruct bone in the maxillofacial region but also increase the success rates of dental implants and other restorative procedures, ultimately leading to better patient chewing, improved aesthetics, and enhanced quality of life.

Nevertheless, it is important to note that despite the use of aseptic techniques during the harvesting and transplantation of bone grafts, the risk of contamination persists. Microbiotas can develop on the material between the time of harvesting and transplantation [34]. Research by S. Ghanaati et al. [35], which analyzed allogenic bone blocks available on the market, found that three out of five materials were contaminated with organic and cellular residues. J. Lorenz et al. [36] conducted histological analysis of the Maxgraft material, uncovering cellular debris, intertrabecular fat, connective tissue, and collagen. In a study by T. Fretwurst et al. [37], evaluations of allografts from various donors showed that the cleaning techniques used for bone blocks did not fully eliminate contaminants. Further histological examinations by Lorenz J. et al. [38] of allogeneic bone blocks implanted in patients revealed the presence of donor cellular remnants; however, these remnants did not adversely affect alveolar process regeneration. These findings suggest that more effective cleaning and decontamination methods for graft materials should be adopted by both manufacturers and clinicians.

Although the number of studies identified by our search is relatively small, the studies present a variety of outcomes. During this decade, laboratory studies, experimental models, and clinical trials have been conducted, providing data on the morphology of ground dentin and the composition of the dentin material and showcasing how the graft integrates with adjacent tissues providing support for implants. Clinical studies used demineralized dentin grafts in the following indications: the regeneration of alveolar bone and the prevention of its loss after extraction [14,15,17,23,26,27], the sinus lift procedure [24], and unilateral alveolar cleft [25]. The regeneration of alveolar bone has not been extensively studied in patients with specific pathologies and has primarily been explored in small patient populations. Furthermore, these indications are currently limited to dentistry and maxillofacial surgery, reflecting a natural progression toward using extracted teeth as a material in maxillofacial procedures. Although typically demineralized dentin is not considered a suitable material for skeletal bone regeneration due to the scarcity of autologous grafting material, it holds potential as a source of growth factors for bone regeneration [39]. It is worth noting that, while there is scientific interest and ongoing research in this area, the use of dentin for regenerating bones other than the alveolar bone is far from being a standard clinical practice. Clinical trials and further research are necessary to determine the safety and efficacy of such approaches before they can be widely adopted.

A literature gap has been identified, revealing a lack of emphasis on periprocedural and long-term complications in the existing studies. Patient-reported outcomes were notably absent, and the evaluation of satisfaction has not been conducted, underscoring the need for further research in these areas. The strength of this review lies in its focus on a specific device. Dłucik et al. [24] demonstrated that grinding with various devices yields dentin particles of varying quality in terms of size and shape. Additionally, dentin preparation protocols significantly impact the osteoinductivity and osteoconductivity of dentin [40]. The limitation of our systematic review was its intentionally focused and narrow scope; however, this approach aimed to cover a sufficient range of study types, providing data on both molecular and clinical aspects, thereby connecting laboratory studies with clinical research. In line with this intention, we also limited the search to papers published in English to avoid potential errors that could arise from misinterpretation due to limited proficiency in other languages. In future research on the use of dentin biomaterial in bone reconstruction, it would be worthwhile to expand the molecular aspect with studies indicating the presence and quantity of pro-inflammatory factors, such as cytokines and chemokines, as well as the hydrophobic enzyme ALP and adhesion proteins, which play a key role in the process of bone regeneration. In addition, the determination of additional growth factors, such as IGF, may also prove important in understanding, at the molecular level, the process of bone regeneration using dentin biomaterial. Additionally, further research should be conducted to enable the synthesis of the obtained results through meta-analysis.

## 5. Conclusions

In conclusion, dentin biomaterial emerges as a novel and versatile bone substitute, demonstrating high biocompatibility and ease of acquisition. The preservation of its internal structure, enriched with substantial organic matter, enhances its potential for effective bone regeneration. Particularly, in dental surgery, materials derived from teeth, with inherent growth factors, present a promising alternative to traditional bone autografts, offering the potential to reduce patient morbidity and treatment costs. Examining the molecular aspects of bone tissue regeneration with ground dentin provides a comprehensive understanding, paving the way for targeted and optimized approaches in the realm of regenerative medicine and maxillofacial surgery.

## Figures and Tables

**Table 1:** The Population, Intervention, Comparison, Outcomes, and Study Design (PICOS) framework.

	Inclusion Criteria	Exclusion Criteria
Population	Human studies, regardless of the age of the participants, experimental models, and in vitro studies	
Intervention	Dentin biomaterial used for bone regeneration produced with the dentin grinder	Dentin crushed or trimmed by other methods
Comparators	Any procedure of bone regeneration or none	
Outcomes of interest	Any	The technical aspect of material harvesting without investigating its biological activity and molecular aspects
Study designs	Clinical trials involving humans Case reports	Reviews Letters Technical report Proof-of-concept studies

**Table 2:** Characteristics of the included studies.

Author	Study Design	Study Material */Tissue	Outcomes	
			Physicochemical/Molecular	Clinical
Bianchi, 2021 [20]	In vitro; 4 teeth from 1 donor	**Mineralized dentin (human)** Deproteinized and demineralized dentin Demineralized dentin Deproteinized bovine bone Tissue: cell lines: human periodontal ligament fibroblasts	- A statistically significant difference in the proliferation of cells exposed to test materials compared to the control material at specific time intervals: moment of seeding, 24 h after seeding, 72 h after seeding, and 7 days after seeding. - After 24 h exposure of hPLFS cells to TT and DDP, larger polygonal-shaped cells were observed. For SG and BIOS, the cells were large and more fusiform. - After 72 h in all cases analyzed, a higher culture density was observed; the cells were large and polygonal with a high presence of cytoplasmic processes within the exposure to the test material. - After 7 days, cells exposed to DDP and TT still showed a large polygonal shape; in addition, cells in the TT group showed small white particles inside the body. - In all samples, cell nuclei were oval or rounded in shape. - Fibroblasts exposed to different materials showed expression of vinculin, integrins, and actin to varying degrees at different times of observation. - Specific morphology of dentin materials—the presence of dentinal tubules. The surface of the DDP appeared smoother compared to the SG; the surface of the TT was irregular and jagged. BIOS showed an irregular surface of mineralized bone.	- None.
Khanijou et al., 2021 [21]	In vitro; 12 teeth	**Tooth-derived bone substitute (human)** Allografts (OraGRAFT, DO BONE) Xenograft (BioOss) Alloplast (BoneCeramic) Human mandibular ramus bone Tissue: cell lines: human fetal osteoblastic cells	- After chemical treatment, the dental tubules became visible and appeared homogeneous. - The tooth-derived bone substitute (TDBS) contained O (50.59%), C (21.98%), Ca (15.71%), P (9.82%), Na (1.36%), and Mg (0.55%). - The Ca/P ratio for TDBS was 1.60 and was similar to Bio-Oss and the mandibular bone. - TDBS contained hydroxylapatite, octacalcium phosphate similar to Orograft and the mandibular bone, and Bio-Oss^®^ additionally contained tricalcium phosphate, while boneceramic^®^ contained only hydroxylapatite and tricalcium phosphate. - TDBS and the mandibular bone showed a gradual increase in Ca ion dissociation over time; in contrast, oragraft Ca ions showed intense dissociation early on, while Bio-Oss and boneceramic had lower total calcium release. - The hFOB cells migrated significantly more in the presence of TDBS; moreover, on days 5 and 10, TDBS showed higher BMP2 release than allograft. - The absence of bacteria in dentin after treatment with the cleaning agent.	- None.
Sarna-Boś et al., 2022 [22]	In vitro; 50 teeth 50 donors	**Four groups (incisors, canines, premolars, molars) crushed without chemical processing**	- All tooth types contained similar proportions of amide I, amide II, carbonate, and phosphate ions. Premolar teeth had the highest content of the analyzed functional groups, while incisors and canines had the lowest. The content of aliphatic structures was highest in incisors and lowest in premolars. Hydroxyapatite crystallinity showed the following trend: CI: premolars > molars > canines > incisors. - The highest number of negatively dissociating functional groups was observed in molars and the lowest in incisors and canines. - The mean Ca, P, and Na contents were the highest in molars and Mg in canines. The Ca/P ratio was 1.89 for incisors and molars, while it was 1.86 for canines and 1.85 for premolars. - All the tooth types analyzed had a similar percentage elemental composition (O, C, Ca, N, P, Na, Mg). - The pore area was 1.89 m^2^/g and did not exceed 29% of the tooth surface.	- None.
Binderman et al., 2014 [15]	2 cases	**Autogenous mineralized dentin matrix** Tissue: alveolar bone regeneration after extraction	- Integration of the newly formed bone matrix with the transplanted dentin. - No bacterial growth in the dentin after application of the cleaning agent: 0.5 M NaOH and 30% alcohol (*v*/*v*).	- Successful and stable anchorage of the implant.
Cervera-Maillo et al., 2021 [23]	10 patients	**Autologous dentin** with platelet-rich plasma Tissue: alveolar bone after tooth extraction	- As time passed, residual graft and connective tissue decreased and new bone formation increased.	- None.
De Biase et al., 2020 [17]	Split-month case report	**Autologous demineralized dentin matrix** Tissue: alveolar bone regeneration after extraction	- None.	- 3 months after surgery, a reduction in the distal pocket of the second molar at the test site was observed from 4 mm on the day of surgery to 3 mm; no change was observed at the control site (4 mm on the day of surgery and after 3 months).
Del Canto-Díaz et al., 2019 [27]	9 patients; split-mouth study	**Autologous dental material** Unfilled extraction sockets Tissue: alveolar bone regeneration after extraction	- Significant differences in density between the control group and the ADM group of the coronal alveolar and medial alveolar areas both immediately after surgery, at 8 weeks, and at 16 weeks. - Density between the control group and the ADM group in the apical alveolar area immediately after surgery and after 8 weeks was statistically significantly different. - The Ca/P ratio in dentin is similar to that in bone.	- In the control group, the VL distance decreased by 1.77 mm after 16 weeks, while in the autologous dental material (ADM) group, it decreased by 0.42 mm. - The HL-BCB distance after 16 weeks in the control group decreased by 2.22 mm in the buccal cortical area, and the ADM group had a resorption of 0.16 mm. - Significant differences in loss of VL-BCB distance, especially at 1 and 3 mm.
Dłucik et al., 2023 [24]	21 patients in the SDG group	**Autologous demineralized dentin matrix** Tissue: alveolar bone regeneration after extraction, Sinus lift procedure	- The main elemental components of dentin are C, O, P, and Ca. - The material obtained with the bonmaker contained mainly C, O, and small amounts of N, Mg, and Si. The material obtained was characterized by homogeneous size, irregular edges, and the presence of dental tubules. - The tooth transformer material was predominantly O, C, P, and Ca, with particles of varying sizes, smooth surfaces, and visible tubules. - The material obtained with the dentin grinder contained the highest concentration of Ca, similar to dentin, and was the most similar to dentin in terms of elemental composition.	- No complications during the convalescence period. - Implantation is possible after approx. 3 months in the mandible and approx. 4 months in the maxilla. - No loss or increase in the vertical and horizontal dimensions of the alveolar ridge 3–4 months after implantation in bone enriched with the demineralized dental matrix (DDM) and dental matrix (DM); dimensions stable for at least 6 months. - Similar results of the bone-strengthening procedure and implantation for each of the devices analyzed (bonmaker, tooth transformer, dentin grinder).
Dłucik et al., 2023 [28]	13 patients in the SDG group	**Autologous demineralized dentin matrix** Tissue: alveolar bone regeneration after extraction	- None.	- Pooled efficacy and safety for several devices. - Complications.
Matsuzawa et al., 2022 [25]	Case report	**Autologous demineralized dentin matrix** (primary teeth) Tissue: unilateral alveolar cleft	- Six months after transplantation, the demineralized dentin matrix (DDM) differed from the original bone on radiographs in terms of structure and radioresistance. - No visible DDM 2 years after surgery.	- None.
Pohl et al., 2020 [26]	12 patients; 58 sockets	**Autologous mineralized dentin** with platelet-rich plasma Tissue: alveolar bone after tooth extraction	- After 4 months, there was a decrease in ridge width and an increase in buccal and lingual bone height. - The formation of a new bone in direct contact with dentin (ankylosis). - The presence of osteoblasts and pre-osteoblasts between the dentin particles and the newly formed bone.	- No post-transplant complications after 4 months. - No inflammation or fibrous encapsulation around dentin.
Santos et al., 2021 [14]	52 patients; 66 implants	**Autogenous mineralized dentin matrix** Xenograft granules	- Implants placed in sites treated with a mineralized dentin matrix (MDM) had similar stability, bone morphology, and gingival keratinized compared to sites treated with xenograft granules. - MDM-treated sites had a significantly higher percentage of newly formed bone tissue and a lower percentage of residual graft material compared to the xenograft group.	- On the first day after surgery, there was a significantly higher level of pain in the control group, but seven days after surgery, both the MDM and xenograft groups had similar levels of pain and discomfort.
Jun et al., 2014 [29]	38 patients; 19 Bio-Oss; 19 AutoBT	**Autogenous tooth bone graft (AutoBT)** Xenograft (BioOss)	- Osteoid thickness in the Bio-Oss group is 8.35 µm and 13.12 µm in the AutoBT group. - Trabecular thicknesses in the Bio-Oss group is 0.07 µm and 0.08 µm in the AutoBT group.	- None.

ADM, autologous dental material; BCB, buccal cortical bone; DG, dentin grinder; hFOB; human fetal osteoblastic cell; hPLF, human periodontal ligament fibroblast; MDM, mineralized dentin matrix; TDBS, tooth-derived bone substitute; TT, demineralized dentin. * Tooth-derived material processed with a dentin grinder marked in bold.

**Table 3:** Risk of bias evaluation of the included studies.

**Case Reports**	**Binderman [15]**	**De Biase [17]**	**Matsuzawa [25]**
Were the patient’s demographic characteristics clearly described?	No	Yes	Yes
Was the patient’s history clearly described and presented as a timeline?	No	Yes	Yes
Was the current clinical condition of the patient on presentation clearly described?	Yes	Yes	Yes
Were diagnostic tests or assessment methods and the results clearly described?	Yes	Yes	Yes
Was the intervention(s) or treatment procedure(s) clearly described?	Yes	Yes	Yes
Was the post-intervention clinical condition clearly described?	Yes	Yes	Yes
Were adverse events (harms) or unanticipated events identified and described?	Yes	No	Yes
Does the case report provide takeaway lessons?	Yes	No	Yes
**Case Series**			**Pohl [26]**
Were there clear criteria for inclusion in the case series?			Yes
Was the condition measured in a standard, reliable way for all participants included in the case series?			Yes
Were valid methods used for the identification of the condition for all participants included in the case series?			Yes
Did the case series have consecutive inclusion of participants?			Yes
Did the case series have a complete inclusion of participants?			Yes
Was there clear reporting of the demographics of the participants in the study?			No
Was there clear reporting of the clinical information of the participants?			Yes
Were the outcomes or follow-up results of cases clearly reported?			Yes
Was there clear reporting of the presenting site(s)/clinic(s) demographic information?			Yes
Was statistical analysis appropriate?			Yes
**Cohort Studies**			**Cervera-Maillo [23]**
Were the two groups similar and recruited from the same population?			Yes
Were the exposures measured similarly to assign people to both exposed and unexposed groups?			Yes
Was the exposure measured in a valid and reliable way?			Yes
Were confounding factors identified?			No
Were strategies to deal with confounding factors stated?			No
Were the groups/participants free of the outcome at the start of the study (or at the moment of exposure)?			Yes
Were the outcomes measured in a valid and reliable way?			Yes
Was the follow-up time reported and sufficient to be long enough for outcomes to occur?			Yes
Was the follow up complete, and if not, were the reasons for the loss of follow-up described and explored?			Yes
Were strategies to address incomplete follow-up utilized?			NA
Was appropriate statistical analysis used?			Yes
**Randomized Controlled Trials**		**Santos [14]**	**Jun [29]**
Was true randomization used for the assignment of participants to treatment groups?		Yes	Yes
Was allocation to treatment groups concealed?		Yes	Unclear
Were treatment groups similar at the baseline?		Yes	Yes
Were participants blind to treatment assignment?		Yes	Unclear
Were those delivering the treatment blind to treatment assignment?		No	Unclear
Were treatment groups treated identically other than the intervention of interest?		Yes	Yes
Were outcome assessors blind to treatment assignment?		Yes	Unclear
Were outcomes measured in the same way for treatment groups?		Yes	Yes
Were outcomes measured in a reliable way?		Yes	Yes
Was follow-up complete, and if not, were differences between groups in terms of their follow-up adequately described and analyzed?		Yes	Unclear
Were participants analyzed in the groups to which they were randomized?		Yes	Yes
Was appropriate statistical analysis used?		Yes	Yes
Was the trial design appropriate and were any deviations from the standard RCT design accounted for in the conduct and analysis of the trial?		Unclear	Unclear

## Data Availability

Data supporting the findings of this study are available within the article.
